# Wnt/β-catenin signalling activates IMPDH2-mediated purine metabolism to facilitate oxaliplatin resistance by inhibiting caspase-dependent apoptosis in colorectal cancer

**DOI:** 10.1186/s12967-024-04934-0

**Published:** 2024-02-03

**Authors:** Yuting Huang, Szehoi Chan, Shuna Chen, Xueqi Liu, Miao Li, Liyuan Zheng, Zhaoxia Dong, Ziyi Yang, Zixuan Liu, Disheng Zhou, Xingding Zhang, Bo Zhang

**Affiliations:** 1 Department of Interventional Center, The Sixth Affiliated Hospital, Sun Yat-Sen University, Guangzhou, China; 2 Biomedical Innovation Center, The Sixth Affiliated Hospital, Sun Yat-Sen University, Guangzhou, China; 3 Shenzhen Key Laboratory for Systems Medicine in Inflammatory Diseases, School of Medicine, Shenzhen Campus of Sun Yat-Sen University, Sun Yat-Sen University, Shenzhen, China; 4 Department of Dermatovenereology, The Seventh Affiliated Hospital, Sun Yat-Sen University, Shenzhen, China; 5 Dongguan Key Laboratory of Medical Bioactive Molecular Developmental and Translational Research, Guangdong Provincial Key Laboratory of Medical Molecular Diagnostics, Guangdong Medical University, Dongguan, China

**Keywords:** Colorectal cancer, Oxaliplatin resistance, Purine metabolism, IMPDH2, β‑catenin, Apoptosis

## Abstract

**Background:**

Oxaliplatin resistance usually leads to therapeutic failure and poor prognosis in colorectal cancer (CRC), while the underlying mechanisms are not yet fully understood. Metabolic reprogramming is strongly linked to drug resistance, however, the role and mechanism of metabolic reprogramming in oxaliplatin resistance remain unclear. Here, we aim to explore the functions and mechanisms of purine metabolism on the oxaliplatin-induced apoptosis of CRC.

**Methods:**

An oxaliplatin-resistant CRC cell line was generated, and untargeted metabolomics analysis was conducted. The inosine 5ʹ-monophosphate dehydrogenase type II (IMPDH2) expression in CRC cell lines was determined by quantitative real-time polymerase chain reaction (qPCR) and western blotting analysis. The effects of IMPDH2 overexpression, knockdown and pharmacological inhibition on oxaliplatin resistance in CRC were assessed by flow cytometry analysis of cell apoptosis in vivo and in vitro.

**Results:**

Metabolic analysis revealed that the levels of purine metabolites, especially guanosine monophosphate (GMP), were markedly elevated in oxaliplatin-resistant CRC cells. The accumulation of purine metabolites mainly arose from the upregulation of IMPDH2 expression. Gene set enrichment analysis (GSEA) indicated high IMPDH2 expression in CRC correlates with PURINE_METABOLISM and MULTIPLE-DRUG-RESISTANCE pathways. CRC cells with higher IMPDH2 expression were more resistant to oxaliplatin-induced apoptosis. Overexpression of IMPDH2 in CRC cells resulted in reduced cell death upon treatment with oxaliplatin, whereas knockdown of IMPDH2 led to increased sensitivity to oxaliplatin through influencing the activation of the Caspase 7/8/9 and PARP1 proteins on cell apoptosis. Targeted inhibition of IMPDH2 by mycophenolic acid (MPA) or mycophenolate mofetil (MMF) enhanced cell apoptosis in vitro and decreased in vivo tumour burden when combined with oxaliplatin treatment. Mechanistically, the Wnt/β-catenin signalling was hyperactivated in oxaliplatin-resistant CRC cells, and a reciprocal positive regulatory mechanism existed between Wnt/β-catenin and IMPDH2. Blocking the Wnt/β-catenin pathway could resensitize resistant cells to oxaliplatin, which could be restored by the addition of GMP.

**Conclusions:**

IMPDH2 is a predictive biomarker and therapeutic target for oxaliplatin resistance in CRC.

**Supplementary Information:**

The online version contains supplementary material available at 10.1186/s12967-024-04934-0.

## Introduction

Colorectal cancer (CRC) is the third most commonly diagnosed cancer and the second most common cause of cancer-related death worldwide, and patients are being diagnosed at a younger age and more advanced stage, although its annual incidence and mortality rate continue to decline [1]. Oxaliplatin is currently one of the most effective chemotherapeutic drugs for CRC treatment, and oxaliplatin-based combination chemotherapy regimens, such as FOLFOX, CapeOX and FOLFOXIRI, are widely used as first-line treatments in the clinic [2]. However, accumulating research has indicated that 40 – 50% of CRC patients will develop drug resistance before or during oxaliplatin treatment, which leads to therapeutic failure and poor prognosis [3]. The mechanisms of oxaliplatin resistance, a multifactorial phenomenon, have only been partially described [4]. Therefore, understanding the mechanisms of oxaliplatin resistance and seeking effective strategies to reverse resistance are critical to overcome such challenges and improve patient survival.

Metabolic reprogramming, a hallmark of cancer, is a key contributor to tumour progression and therapeutic resistance [5]. Metabolic rate-limiting enzymes, which regulate the direction and speed of metabolic pathways, play critical roles in metabolic reprogramming [6] Strategies that target aberrant metabolism and key metabolic enzymes have great potential as therapeutic options for enhancing the drug susceptibility of cancers [7]. Inosine 5'-monophosphate dehydrogenase (IMPDH) is one of key rate-limiting enzymes in purine metabolism and plays significant biological functions in normal physiological activities and abnormal diseases [8]. As one of two distinct isoforms, IMPDH1 is generally less expressed than IMPDH2 is in most tissues [9] and is the main constitutively expressed protein in normal human leukocytes and lymphocytes [10]. However, IMPDH2 is particularly overexpressed in rapidly proliferating and neoplastic cells [11]. Numerous studies have shown that high IMPDH2 expression is associated with tumorigenesis and progression in most cancers [12, 13], indicating that IMPDH2 may be a significant biomarker [14]. Inhibition of IMPDH2 activity by mycophenolic acid (MPA) suppressed guanosine triphosphate (GTP) synthesis and increased the radiosensitivity of glioblastoma cells by impairing DNA repair [15]. However, whether aberrant cancer metabolism supports oxaliplatin resistance remains unknown.

The Wnt/β-catenin signalling pathway plays vital roles in normal embryonic maintenance and development [16] and can regulate cell proliferation and apoptosis in multiple tumours [17]. Overactive Wnt signalling leads to decreased degradation of β-catenin and increased entry into the nucleus, which, via lymphoid enhancer factor/T-cell factor (LEF/TCF) transcription factors, results in inappropriate activation of downstream target genes, including c-Myc, Cyclin D1, MMP7, etc. [18]. Previous studies suggested that aberrant Wnt/β-catenin signalling pathway is involved mainly in cancer cell proliferation and tumour progression [19], mainly of gastrointestinal origin [20]. A growing body of research now revealed that upregulated Wnt/β-catenin promotes resistance to chemotherapy in multiple cancers [21, 22]. The Wnt/β-catenin pathway can direct glycolysis and blocking Wnt/β-catenin signalling reduces glycolytic metabolism and results in the inhibition of tumour growth and angiogenesis [23, 24]. To date, however, whether the Wnt/β‑catenin pathway affects cancer metabolism and modulates chemoresistance in CRC has not been determined.

In the present study, we investigated the metabolic changes that occur in oxaliplatin-resistant CRC cells and identified increased purine metabolism, which mainly arises from the upregulation of IMPDH2 expression through the Wnt/β‑catenin pathway. IMPDH2 affects resistance to apoptosis induced by oxaliplatin through the Caspase 7/8/9 and PARP1 proteins. These findings suggest that the regulation of purine metabolism by IMPDH2 plays an essential role in oxaliplatin resistance and provide evidence that a novel therapeutic strategy targets IMPDH2 to restore the sensitivity of CRC to oxaliplatin.

## Materials and methods

### Drugs and chemicals

Oxaliplatin (S1224) was purchased from Selleck. Guanylic acid disodium salt (GMP, GC35159) was purchased from GlpBio. Mycophenolic acid (MPA, HY-B0421), Mycophenolate mofetil (MMF, HY-B0199) and XAV939 (HY-15147) were obtained from MedChemExpress.

### Cell lines and culture

The human CRC cell lines SW620, RKO, HCT116 and HCT8 were obtained from the Cell Bank of the Chinese Academy of Sciences (Shanghai, China). HEK293T cells were obtained from the ATCC (USA). Oxaliplatin-resistant HCT8/L-OHP cells were established by continuously culturing parental HCT8 cells in the presence of stepwise increasing concentrations of oxaliplatin over approximately 10 months. SW620, HCT116, HCT8 and HCT8/L-OHP cells were cultured in RPMI-1640 medium (Gibco, USA), while RKO and HEK293T cells were grown in high-glucose DMEM (Gibco, USA). All culture media contained 10% foetal bovine serum (Hyclone, USA), 100 U/mL penicillin, and 100 µg/mL streptomycin (Gibco, USA). All cell lines were maintained in a humidified chamber with 5% CO_2_ at 37 ℃.

### Establishment of oxaliplatin-resistant cells

To establish oxaliplatin-resistant cells, parental HCT8 cells were initially cultured in RPMI-1640 medium supplemented with 1 µmol/L oxaliplatin for 48 h. The drug concentration was increased by 2 µmol/L once the cells recovered to normal growth after 2 weeks of continuous drug exposure. The 50% inhibitory concentration (IC50) of oxaliplatin at each dose was determined by a CCK-8 assay, and the resistance index (RI) of the oxaliplatin-resistant cells was defined as follows: RI = IC50 of resistant cells/IC50 of parental cells. Stable oxaliplatin-resistant cells were selected and maintained at a final drug concentration of 25 µmol/L after approximately 10 months, and the induced oxaliplatin-resistant cells were named HCT8/L-OHP. Single cell-derived clones of HCT8/L-OHP cells were obtained by a limiting dilution strategy and were used for further study. For all experiment, the HCT8/L-OHP cells were refreshed with oxaliplatin-free medium for 48 h before treatment.

### Flow cytometry analysis of cell apoptosis

Cells were seeded at 2 × 10^5^ cells per well into 12-well plates with three duplicate wells. After 24 h of incubation, the cells were treated with different concentrations of oxaliplatin for another 24 h. Subsequently, the cells were washed with PBS, harvested using trypsin without EDTA, resuspended in binding buffer and stained with Annexin V-APC/PI (Multi Sciences, AP107) for 15 min in the dark according to the manufacturer's protocols. The percentage of apoptotic cells was measured using a CytoFLEX flow cytometer (Beckman Coulter) and analysed using CytExpert software (Beckman Coulter). The results are presented as the percentage of total cells which were living cells, early apoptotic cells, late apoptotic cells and necrotic cells.

### Overexpression and knockdown of IMPDH2 in CRC cells

For ectopic overexpression of IMPDH2, the coding sequence of Flag-tagged IMPDH2 was amplified via PCR and inserted into the pcDNA3.1 (+) vector. HCT8 and SW620 cells were transiently transfected using Lipofectamine^™^ 3000 (Invitrogen, L3000015) according to the manufacturer's instructions. After 24 or 48 h of transfection, the following series of experiments were performed.

To establish stable IMPDH2-knockdown cells, the short hairpin RNA (shRNA) for IMPDH2 was cloned and inserted into the pLKO.1-puromycin lentiviral vector. Then HEK293T cells were cotransfected with the psPAX2 packaging plasmid and pMD2.G envelope plasmid using Lipofectamine^™^ 3000 to produce lentivirus. After transfection for 48 h, the viral supernatants were collected and used to infect 5 × 10^5^ HCT8/L-OHP cells after filtration, followed by selection with 2 µg/mL puromycin selection. The IMPDH2 knockdown efficiency was verified by qPCR and western blotting analysis. The sequences of IMPDH2-shRNA were as follows: IMPDH2-shRNA-F: 5ʹ-CCGGCGGAAAGTGAAGAAATATGAACTCGAGTTCATATTTCTTCACTTTCCGTTTTTG-3ʹ.

### CCK-8 assay

For the CCK-8 assay, 6 × 10^3^ cells in 100 µL culture medium were seeded in a 96-well plate with three duplicate wells and incubated for 24 h. Then the cells were treated with various concentrations of oxaliplatin. After 48 h of treatment, 10 µL of the CCK-8 reagent (A311-02) was added to each well and the 96-well plate was subsequently incubated at 37 ℃ for 2 or 4 h. The optical density (OD) value at 450 nm was assessed to determine the inhibition ratio of the cells with an EPOCH spectrophotometer (BioTek Instruments). All the experiments were performed with at least three replicates.

### Total RNA extraction, reverse transcription and quantitative PCR (qPCR)

Total RNA was extracted from cells using TRIzol (Invitrogen, 15596018) according to the manufacturer’s instructions. The extracted total RNA was measured on a NanoDrop spectrophotometer (NanoDrop Technologies, USA) and 500 ng of RNA was reverse transcribed to cDNA using a reverse transcription kit (Takara, RR047A). QPCR was performed using SYBR Green reagent (Takara TB Green, RR420A), and the mRNA level was detected by an ABI Step-One Detection System. Each sample was analysed in triplicate. The relative mRNA expression levels were measured using the 2^−ΔΔCt^ method and β-actin was used as the internal reference. Primers sequence of qPCR were listed in Table 1.

**Table 1:** Primers Sequence of qPCR used in this study

Gene symbol	Primers sequence
PRPS1	Forward: 5ʹ-ATCTTCTCCGGTCCTGCTATT-3′
	Reverse: 5ʹ-TGGTGACTACTACTGCCTCAAA-3ʹ
PRPS2	Forward: 5ʹ -AGCTCGCATCAGGACCTGT-3ʹ
	Reverse: 5ʹ-ACGCTTTCACCAATCTCCACG-3ʹ
PPAT	Forward: 5ʹ-GATGGGAGTTCGGTGCCAA-3ʹ
	Reverse: 5ʹ-CAACGAAGGGCTGACAATTTTC-3ʹ
IMPDH1	Forward: 5ʹ-TGAAGAAGAACCGAGACTACCC-3ʹ
	Reverse: 5ʹ-TCCAGACGGTATTTGTCATCCT-3ʹ
IMPDH2	Forward: 5ʹ-AGGGAAAGTTGCCCATTGTAAA-3ʹ
	Reverse: 5ʹ-TGGGTAGTCCCGATTCTTCTTC-3ʹ
GMPS	Forward: 5ʹ-ATGGCTCTGTGCAACGGAG-3ʹ
	Reverse: 5ʹ-CCTCACTCTTCGGTCTATGACT-3ʹ
ADSS1	Forward: 5ʹ-AAGAAGGGAATCGGACCAACC-3ʹ
	Reverse: 5ʹ-CCGTGGAGTGCCTCATACATAA-3ʹ
ADSS2	Forward: 5ʹ-TGGGTATGCCACCTCAAAATG-3ʹ
	Reverse: 5ʹ-GCTCTGTAGGAAAGGCACCAATA-3ʹ
ADSL	Forward: 5ʹ-GCTGGAGGCGATCATGGTTC-3ʹ
	Reverse: 5ʹ-TGATAGGCAAACCCAATGTCTG-3ʹ
APRT	Forward: 5ʹ-GGCCGCATCGACTACATCG-3ʹ
	Reverse: 5ʹ-CTCAGCCTTCCCGTACTCC-3ʹ
HPRT1	Forward: 5ʹ-ACCAGTCAACAGGGGACATAA-3ʹ
	Reverse: 5ʹ-CTTCGTGGGGTCCTTTTCACC-3′
ADAR	Forward: 5ʹ-CTGAGACCAAAAGAAACGCAGA-3ʹ
	Reverse: 5ʹ-GCCATTGTAATGAACAGGTGGTT-3′
β-catenin	Forward: 5′-AAAGCGGCTGTTAGTCACTGG-3′
	Reverse: 5ʹ-CGAGTCATTGCATACTGTCCAT-3ʹ
c-Myc	Forward: 5ʹ-GGCTCCTGGCAAAAGGTCA-3ʹ
	Reverse: 5ʹ-CTGCGTAGTTGTGCTGATGT-3ʹ
Cyclin D1	Forward: 5ʹ-GAACACGGCTCACGCTTAC-3ʹ
	Reverse: 5ʹ-CCCAGACCCTCAGACTTGC-3ʹ
β-actin	Forward: 5ʹ-CATGTACGTTGCTATCCAGGC-3ʹ
	Reverse: 5ʹ-CTCCTTAATGTCACGCACGAT-3ʹ

### Western blotting analysis

Cell lysates for western blotting were collected in RIPA lysis buffer containing protease inhibitor cocktail (Thermo, 78,443) for 30 min on ice and centrifuged at 12,000 × *g* for 15 min at 4 ℃ to obtain the supernatant. Protein quantification was performed with the Bradford Protein Assay Kit (Thermo, 23,236). Proteins were separated by SDS‒PAGE electrophoresis and subsequently transferred to PVDF membranes (Millipore, ISEQ00010). Then the membranes were blocked with 5% skim milk (BD Difco, 232,100) in Tris-buffered saline containing 0.5% Tween-20 for 1 h and incubated with primary antibodies at 4 ℃ overnight. The primary antibodies included IMPDH2 (1:5000 dilution, #ab131158, Abcam), Caspase 3/p17/p19 (1:3000 dilution, #66470-2-Ig, Proteintech), Caspase 7/p20 (1:1000 dilution, #27155-1-AP, Proteintech), Caspase 8/p43/p18 (1:5000 dilution, #66093-1-Ig, Proteintech), Caspase 9/p35/p10 (1:2000 dilution, #66169-1-Ig, Proteintech), Bax (1:10000 dilution, #60267-1-Ig, Proteintech), PARP1 (1:20000 dilution, #66520-1-Ig, Proteintech), β-Catenin (1:1000 dilution, #8480S, Cell Signaling Technology), Cyclin D1 (1:1000 dilution, #2978S, Cell Signaling Technology), C-Myc (1:1000 dilution, #ab32072, Abcam), P-gp (1:1000 dilution, #22336-1-AP, Proteintech), β-actin (1:5000 dilution, #20536-1-AP, Proteintech). After incubation with the appropriate secondary antibodies (anti-rabbit IgG, 1:10000 dilution, #A16098, Thermo; anti-mouse IgG, 1:10000 dilution, #PA1-28555, Thermo). The protein signals were visualized by an enhanced chemiluminescence (ECL) detection kit (NCM Biotech, P10300).

### Untargeted metabolomics analysis

Untargeted metabolomics analysis was conducted by the liquid chromatography-tandem mass spectrometry (LC–MS/MS) technology, using a Waters 2D UPLC (Waters, USA) coupled with a high-resolution mass spectrometer Q Exactive HF (Thermo Fisher Scientific, USA) with a heated electrospray ionization (HESI) source and controlled by the Xcalibur 2.3 software program (Thermo Fisher Scientific, Waltham, MA, USA). Five microlitres of each sample was injected onto a Waters ACQUITY UPLC BEH C18 column (1.7 μm, 2.1 mm × 100 mm, Waters, USA) for chromatographic separation, and the column temperature was maintained at 45 ℃. The mobile phase consisted of 0.1% formic acid (A) and acetonitrile (B) in positive mode, and in negative mode, the mobile phase consisted of 10 mM ammonium formate (A) and acetonitrile (B). The gradient conditions were as follows: 0–1 min, 2% B; 1–9 min, 2%-98% B; 9–12 min, 98% B; 12–12.1 min, 98% B to 2% B; and 12.1–15 min, 2% B. The flow rate was 0.35 mL/min. Data processing was performed using the Compound Discoverer 3.1 (Thermo Fisher Scientific, USA) software. The mass spectrometric settings for positive/negative ionization modes were as follows: spray voltage, 3.8/ − 3.2 kV; sheath gas flow rate, 40 arbitrary units (arb); aux gas flow rate, 10 arb; aux gas heater temperature, 350 ℃; and capillary temperature, 320 ℃. The full scan range was 70–1050 m/z with a resolution of 70,000, and the automatic gain control (AGC) target for MS acquisitions was set to 3e6 with a maximum ion injection time of 100 ms. The top 3 precursors were selected for subsequent MSMS fragmentation with a maximum ion injection time of 50 ms and resolution of 30,000; the AGC was 1e5. The stepped normalized collision energy was set to 20, 40 and 60 eV. The samples were randomized to reduce systematic errors. A quality control (QC) sample containing equal aliquots of all cell samples was interspersed for every 10 samples.

### Immunoprecipitation-mass spectrometry (IP-MS) analysis

LC‒MS/MS was used to identify the differentially expressed proteins in HCT8 and HCT8/L-OHP cells by transfecting cells with Flag-tagged IMPDH2 plasmids. Briefly, total cell lysates from HCT8 and HCT8/L-OHP cells were collected and incubated with anti-DDDDK-tag mAb-magnetic beads (MBL, M185-11R) at 4 ℃ overnight. Then, the beads were separated by a magnetic device. The cell proteins were identified via LC‒MS/MS using a Q Exactive HF X mass spectrometer (Thermo Fisher Scientific, USA). Subsequently, functional annotation analysis and pathway analysis were performed on the final protein identification list.

### Animal experiment

All animal experiments were conducted in accordance with the Animal Ethics Committee of The Sixth Affiliated Hospital, Sun Yat-sen University (IACUC-2023051101) and all relevant ethical regulations were strictly followed. Male BALB/c nude mice aged 4–6 weeks were obtained from Zhejiang Vital River Laboratory Animal Technology Co., Ltd (Zhejiang, China). Approximately 5 × 10^6^ HCT8/L-OHP cells were subcutaneously injected into the right flank in 100 μL of 50% PBS and 50% Matrigel-basement membrane matrix. Once the tumour volume reached 100 mm^3^, the mice were randomly divided into four experimental groups: control, oxaliplatin alone, MMF alone, or a combination of oxaliplatin and MMF. Oxaliplatin was intraperitoneally injected at a dose of 10 mg/kg every 3 days, and MMF was intragastrically administered at a dose of 120 mg/kg twice a day. The tumour volume and body weight were measured every 3 days. The tumour volume was calculated by the formula: Volume = length × width^2^ × 0.5 cm^3^.

### Statistical analysis

All the data are presented as the means ± SDs of at least three independent experiments. The statistical analysis was performed using GraphPad Prism 8 software (San Diego, 265 California), and the statistically significant differences were analysed using two-tailed Student’s t tests. The statistical significance was defined as follows: ns = not significant, *p < 0.05, **p < 0.01 and ***p < 0.001; these data were included in the respective figure legends.

## Results

### Establishment and characterization of the acquired oxaliplatin‑resistant CRC cell line HCT8/L-OHP

**Fig. 1:**
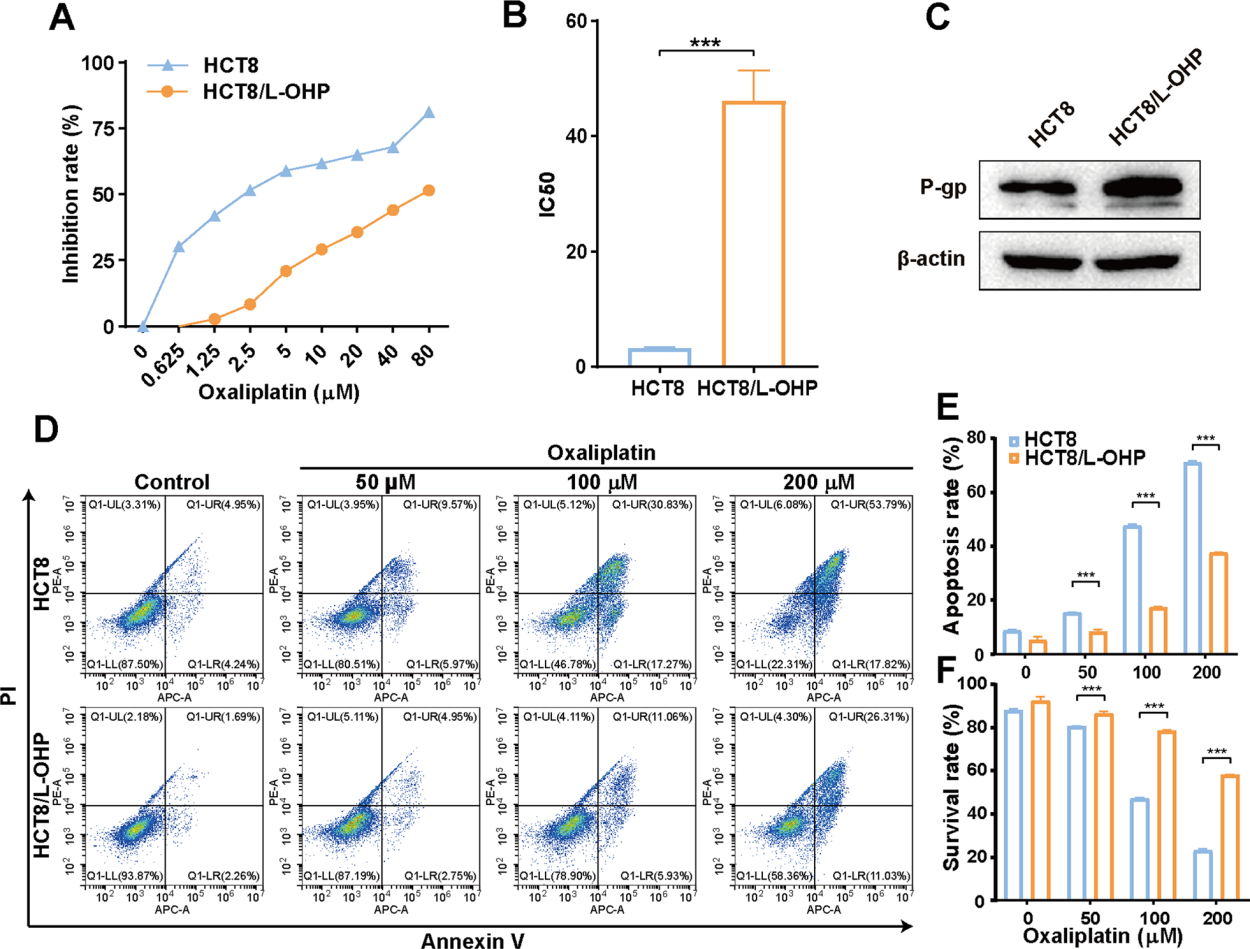
Establishment and characterization of the acquired oxaliplatin-resistant CRC cell line HCT8/L-OHP. **A** CCK8 assay to assess the IC50 of oxaliplatin-resistant CRC cells HCT8/L-OHP and parental HCT8 cells after treated with increasing concentrations of oxaliplatin treatment for 48 h. **B** The IC50 of oxaliplatin to HCT8 and HCT8/L-OHP cells were calculated from the inhibition curves. **C** P-gp protein expression in HCT8 and HCT8/L-OHP cells was determined by western blotting analysis. **D** Flow cytometry analysis measures the apoptosis in HCT8 and HCT8/L-OHP cells at 24 h after oxaliplatin treatment. Bar charts show the percentage of apoptosis **E** and surviving **F** cells, respectively, in both untreated and oxaliplatin-treated groups. Data are presented as the mean ± SD of three independent experiments. ***p < 0.001

To explore the underlying mechanism of oxaliplatin resistance in CRC, we established the acquired oxaliplatin-resistant CRC cell line HCT8/L-OHP by a stepwise escalation method over approximately 10 months. As shown in Fig. 1A, the dose–response growth curve showed that HCT8/L-OHP cells were significantly more resistant to oxaliplatin than were HCT8 cells. Furthermore, the IC50 of oxaliplatin in HCT8 cells was 3.23 ± 0.21 µmol/L, whereas the IC50 in HCT8/L-OHP cells increased to 46.08 ± 5.27 µmol/L, with the resistantce index (RI) of 14.27 (Fig. 1B). Western blotting analysis revealed that the protein expression of the multidrug-resistant P-glycoprotein (P-gp) was significantly upregulated in HCT8/L-OHP cells compared to that in HCT8 cells (Fig. 1C). Moreover, compared with HCT8/LOHP cells, HCT8 cells displayed a greater apoptosis rate and a greater decreased percentage of surviving cells in response to all concentrations of oxaliplatin (Fig. 1D-F). Taken together, these results confirmed that HCT8/L-OHP cells were significantly resistant to oxaliplatin.

### Increased purine metabolism in response to oxaliplatin resistance

**Fig. 2:**
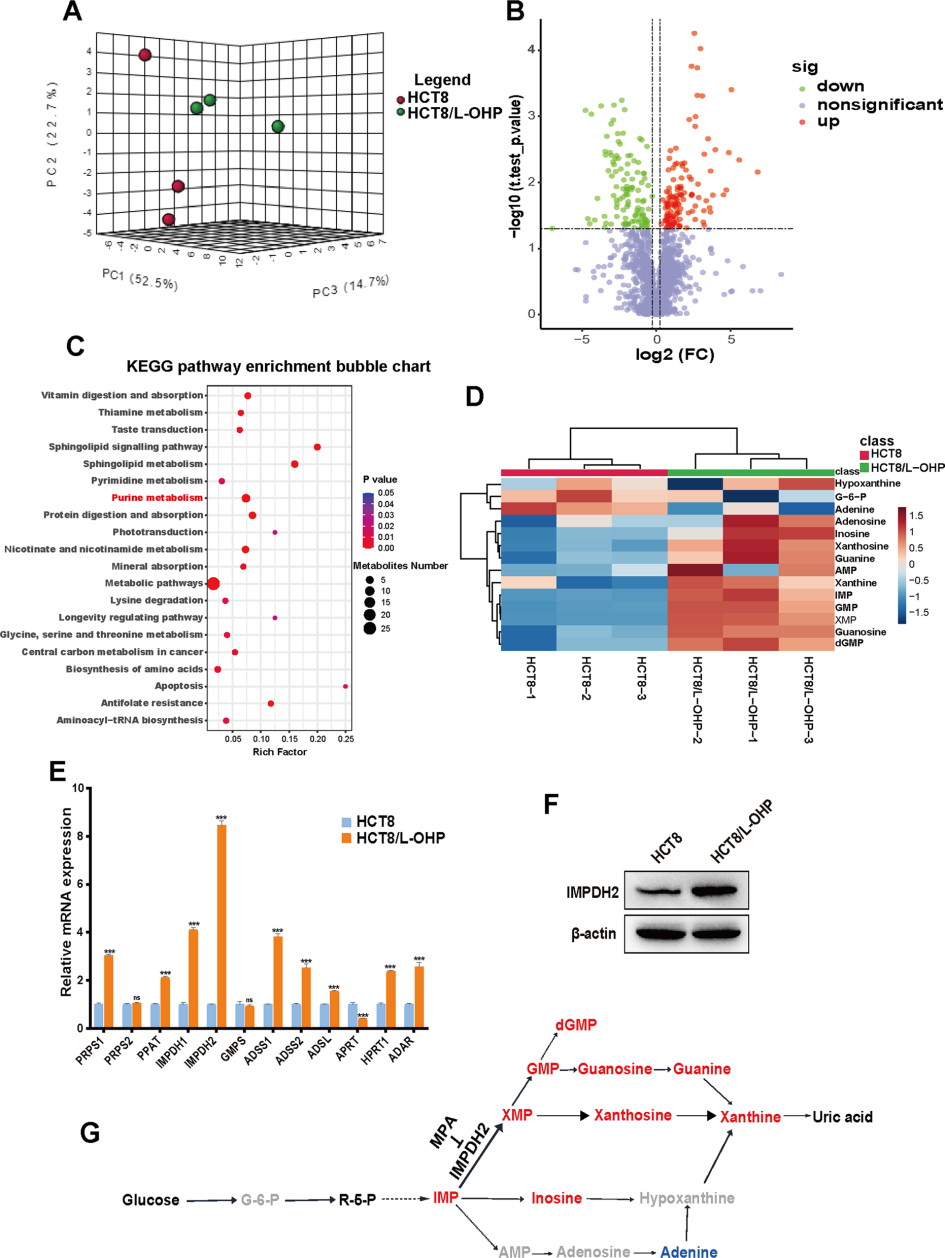
Purine metabolism was upregulated in oxaliplatin-resistant HCT8/L-OHP cells. **A** PCA 3D score plot between HCT8 and HCT8/L-OHP groups. **B** Volcano plot of differential metabolites. **C** Bubble plots for KEGG metabolic pathway enrichment analysis of differential metabolites. **D** Hierarchical clustering analysis of differential metabolites involved in purine metabolism. **E** QPCR analysis of purine metabolic key enzyme genes in HCT8/L-OHP comparing to those in HCT8 cells. ACTB was used as the internal reference. **F** Western blotting analysis of IMPDH2 protein expression in HCT8 and HCT8/L-OHP cells. **G** Schematic diagram of purine metabolism. The red indicates that the metabolite is up-regulated, the blue indicates that the metabolite is down-regulated, while the gray indicates metabolite with no significant change. *G-6-P* Glucose-6-phosphate, *R-5-P* Ribose-5-phosphate, *IMP* Inosinic acid, XMP, Xanthylic acid; GMP, Guanosine monophosphate; dGMP, Deoxyguanosine monophosphate; *AMP* Adenosine monophosphate. Significance is indicated as *ns*  not significant, *p < 0.05, **p < 0.01, and ***p < 0.001

Metabolic reprogramming plays a vital role in tumorigenesis and tumour progression [5]. To investigate the metabolic reprogramming underlying oxaliplatin resistance in CRC, we performed untargeted metabolomics analysis of HCT8 and HCT8/L-OHP cells. As shown in Fig. 2A, significant differences were found between the HCT8 and HCT8/L-OHP groups according to the PCA 3D score-plot clusters. Next, to screen aberrant metabolites and potential signalling pathways, we strictly limited all metabolites shown in the volcano plot (Fig. 2B). Only metabolites with a fold change ≥ 1.2 or ≤ 0.83 and a P value less than 0.05 were selected. In total, 239 metabolites were significantly changed—124 upregulated and 115 downregulated—in HCT8/L-OHP cells. Furthermore, kyoto encyclopedia of genes and genomes (KEGG) pathway enrichment analysis demonstrated that the differentially abundant metabolites were significantly enriched in purine metabolism, as well as sphingolipid metabolism, nicotinate and nicotinamide metabolism and so on (Fig. 2C). We subsequently selected 14 metabolites associated with the purine metabolism map identified in this analysis as candidates. As shown in Fig. 2D and Additional file 1: Figure S1, 9 metabolites had higher levels in HCT8/L-OHP cells than in HCT8 cells, while only one metabolite, adenine, was downregulated. Among the 9 elevated metabolites, the top three were guanosine monophosphate (GMP), deoxyguanosine monophosphate (dGMP) and xanthylic acid (XMP). The metabolites with significant changes are marked in the pathway of the purine metabolism map (Fig. 2G). Metabolic activity is often determined by key rate-limiting enzymes. Thus, we further examined the expression of key metabolic enzymes involved in purine metabolism. Most of the key genes, inculding PRPS1, PPAT, IMPDH1, IMPDH2, ADSS1, ADSS2, ADSL, HPRT1 and ADAR, were significantly upregulated in HCT8/L-OHP cells compared with those in HCT8 cells, especially IMPDH2 (Fig. 2E), which was consistent with the change trend in purine metabolism. The increase in IMPDH2 protein expression in HCT8/L-OHP cells was further confirmed by western blotting analysis (Fig. 2F). These results indicated the activation of purine metabolism in oxaliplatin resistance. Based on these studies, we focused on IMPDH2 in follow-up research.

### High IMPDH2 expression in CRC correlates with activated purine metabolism and multidrug resistance

**Fig. 3:**
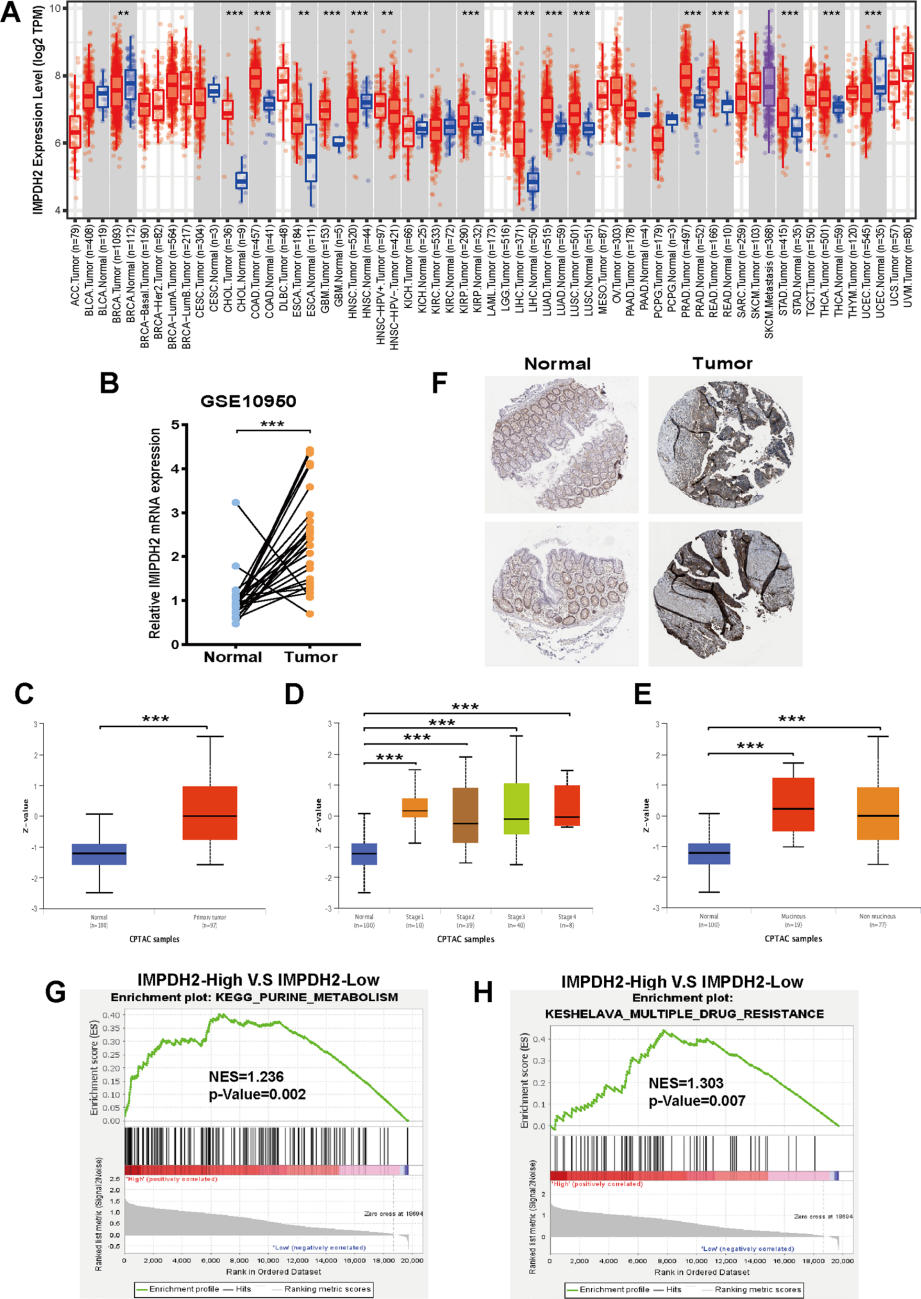
IMPDH2 expression is upregulated in CRC patients and is associated with multiple drug resistance. **A** IMPDH2 expression in various types of cancer in the TIMER database. **B** The mRNA expression of IMPDH2 in 24 matched CRC and adjacent noncancerous samples from the GSE10950. The protein expression of IMPDH2 in CRC based on sample types (**C**), individual cancer stages **D** and histological subtypes **E** from CPTAC. **F** The IMPDH2 protein expression from HPA database. GSEA was performed to showing a significant association between the IMPDH2 expression and the PURINE_METABOLISM **G** and MULTIPLE-DRUG-RESISTANCE **H**. **p < 0.01, and ***p < 0.001

To explore the possible roles of IMPDH2, we first examined the expression of IMPDH2 in 29 types of human tumour tissues and corresponding normal tissues using the TCGA and TIMER databases. The results revealed that the mRNA expression of IMPDH2 was significantly elevated relative to that in normal tissues in several common cancer types, including colon adenocarcinoma (COAD) and rectum adenocarcinoma (READ) (Fig. 3A). Consistently, comparison of IMPDH2 mRNA expression in human colon tumours and paired normal colon mucosa samples from the Gene Expression Omnibus (GEO) databases (GSE10950) demonstrated the upregulation of IMPDH2 in cancer (Fig. 3B). We also used the UALCAN database to evaluate IMPDH2 expression at the protein level. As shown in Fig. 3C–E, the protein expression of IMPDH2 was significantly upregulated in CRC tissues and correlated with stage and histological subtype. In addition, immunohistochemical staining (IHC) analysis of the Human Protein Atlas (HPA) database revealed that the IMPDH2 protein was highly expressed in CRC tissue (Fig. 3F). These results indicated that IMPDH2 is upregulated in CRC.

Furthermore, we also performed gene set enrichment analysis (GSEA) with the TCGA CRC database to identify biological pathways related to high and low IMPDH2 expression. GSEA revealed that the PURINE_METABOLISM gene set was significantly enriched in the IMPDH2-high subgroup (Fig. 3G), consistent with its metabolic function. Interestingly, we found that one enriched gene set, MULTIPLE-DRUG-RESISTANCE, was positively correlated with IMPDH2 expression (Fig. 3H), indicating that IMPDH2 may be involved in multiple drug resistance in CRC. Collectively, these data suggest that IMPDH2 is highly expressed in CRC patients and is associated with multiple drug resistance.

### IMPDH2 expression in CRC cells is negatively correlated with sensitivity to oxaliplatin

**Fig. 4:**
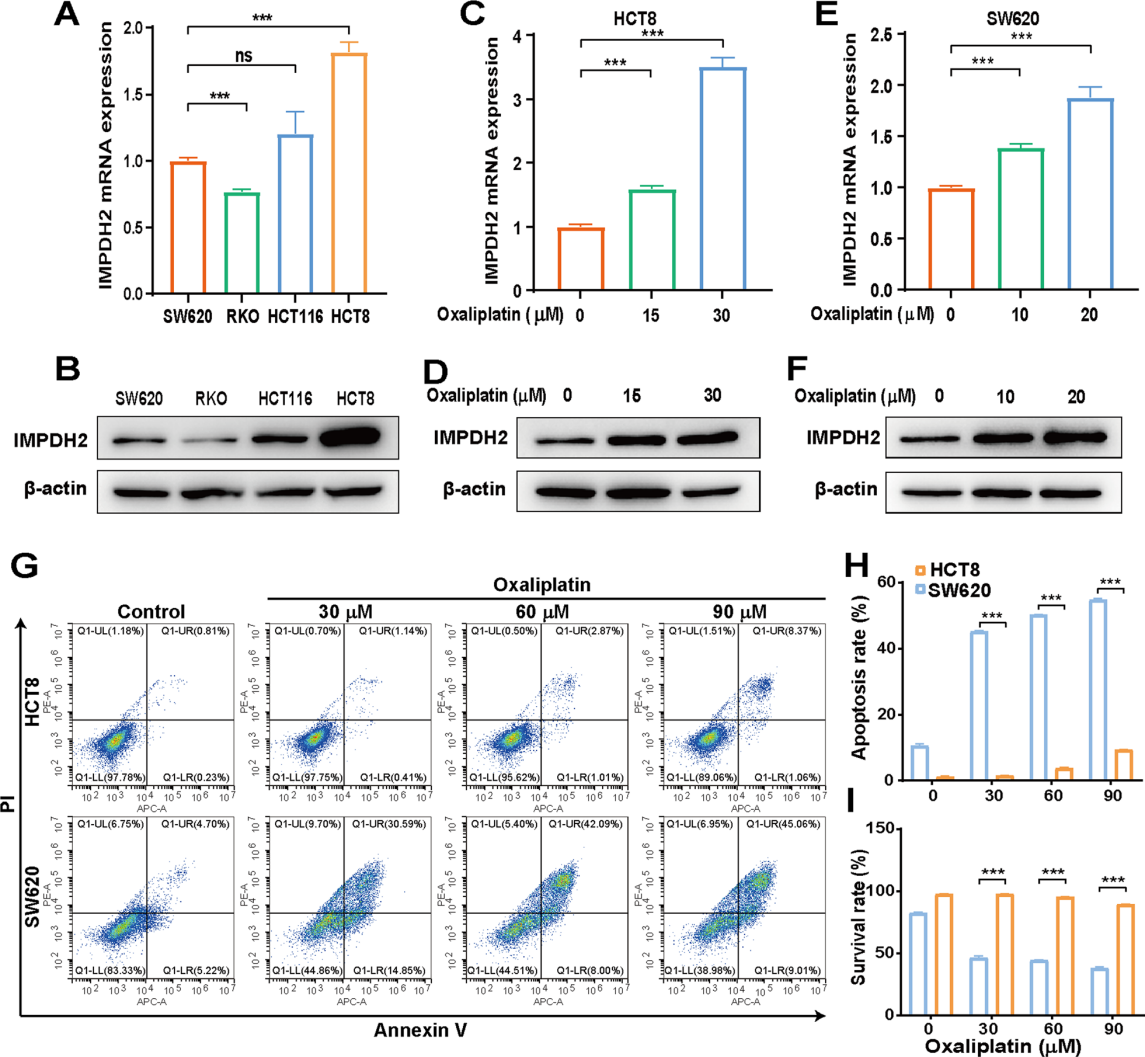
IMPDH2 expression is associated with CRC cells sensitivity to oxaliplatin. **A** and **B** The endogenous expression of IMPDH2 in four CRC cell lines, SW620, RKO, HCT116 and HCT8, was determined by qPCR and western blotting analysis. The mRNA and protein expression of IMPDH2 in HCT8 **C** and **D** and SW620 **E** and **F** in response to oxaliplatin treatment were determined by qPCR and western blotting analysis. **G** Flow cytometric analysis was performed to analyse the apoptotic rate in HCT8 and SW620 cells treated with increasing concentrations of oxaliplatin at 24 h. The histograms show the percentage of apoptosis **H** and surviving **I** cells, respectively, in both untreated and oxaliplatin-treated groups. Data are presented as the mean ± SD of three independent experiments. *ns*  not significant, ***p < 0.001

To further understand the underlying biological function of IMPDH2 in CRC, the expression of endogenous IMPDH2 in four CRC cell lines, SW620, RKO, HCT116 and HCT8, was assessed by qPCR and western blotting analysis. The levels of IMPDH2 expression varied. The mRNA expression of IMPDH2 was significantly greater in HCT8 cells than in RKO and SW620 cells (Fig. 4A). Moreover, western blotting analysis confirmed the markedly increased IMPDH2 protein expression in HCT8 cells but relatively weak in RKO and SW620 cells (Fig. 4B). Next, we treated HCT8 and SW620 cells with different concentrations of oxaliplatin to examine any changes in the expression of IMPDH2. The results indicated that both IMPDH2 mRNA and protein expression could be strongly induced by oxaliplatin in HCT8 (Fig. 4C, D) and SW620 cells (Fig. 4E, F).

To test whether the expression level of IMPDH2 could affect the drug susceptibility of CRC cells to oxaliplatin, HCT8 and SW620 cells were treated with increasing concentrations of oxaliplatin for 24 h to assess cell apoptosis by flow cytometric analysis. Increased percentages of apoptotic cells were observed for both HCT8 and SW620 cells treated with increasing concentrations of oxaliplatin at 24 h after treatment (Fig. 4G). In addition, a greater increase in cell apoptosis was observed in SW620 cells than in HCT8 cells in response to all concentrations of oxaliplatin. For instance, upon 30 µM oxaliplatin treatment, the percentage of total apoptotic cells in SW620 cells was 45.44% (including both early apoptotic cells and late apoptotic cells) but was only 1.55% in HCT8 cells. Increasing the oxaliplatin concentration from 30 to 60 µM resulted in 50.09% of SW620 cells being apoptotic but only 3.88% of HCT8 cells being apoptotic. Moreover, 24 h after oxaliplatin treatment, a significant percentage of cell apoptosis was detected only at a concentration of 90 µM in HCT8 cells, whereas significant cell apoptosis was detected in SW620 cells at all concentrations of oxaliplatin compared to that in the control group (Fig. 4H, I). Therefore, HCT8 cells, which express higher levels of endogenous IMPDH2, were more resistant to oxaliplatin than were SW620 cells, which have markedly lower IMPDH2 expression. These findings suggested that IMPDH2 may be involved in oxaliplatin resistance in CRC.

### IMPDH2-mediated GMP synthesis regulates resistance to apoptosis induced by oxaliplatin through the caspase 7/8/9 and PARP1 proteins

**Fig. 5:**
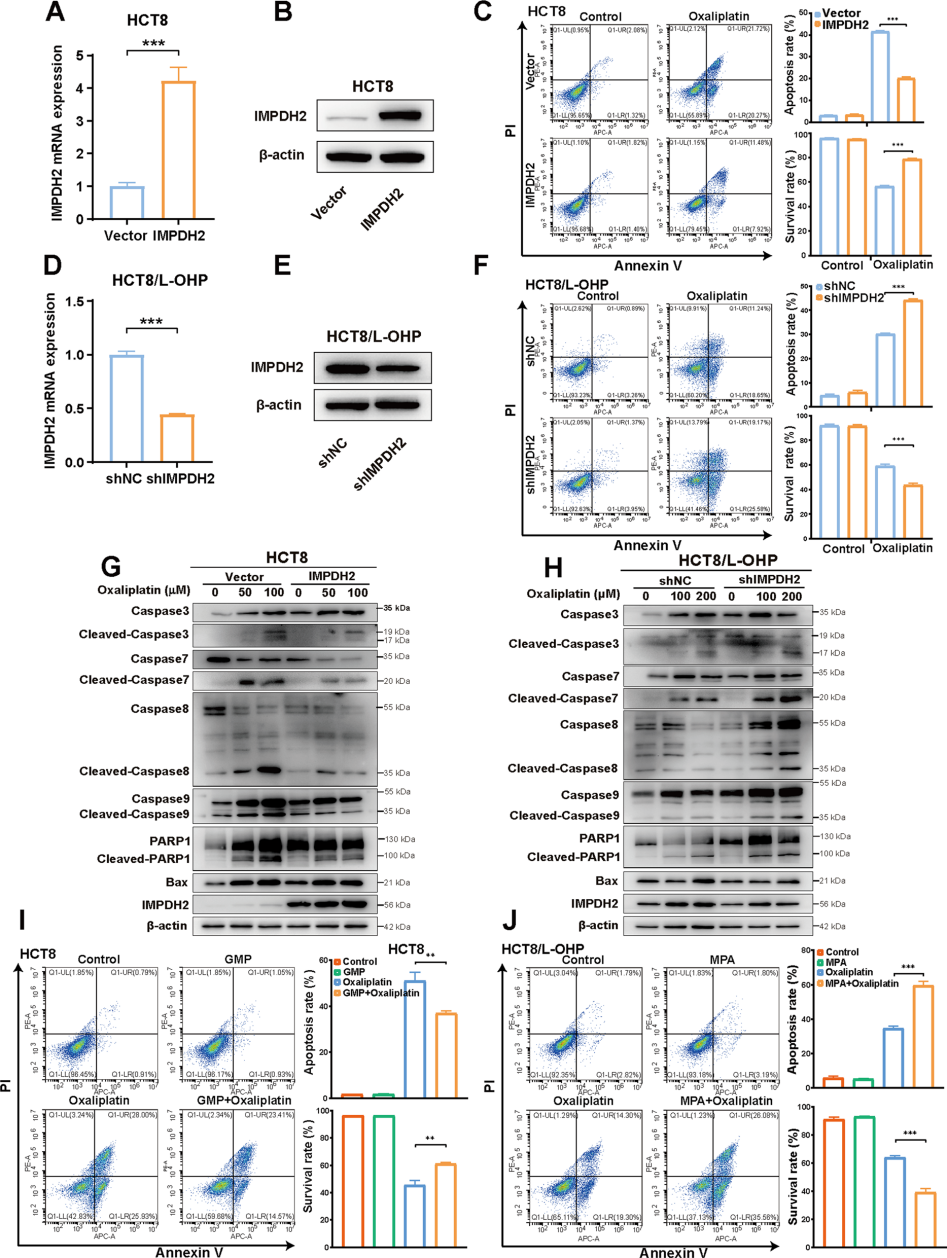
IMPDH2 regulates cell apoptosis of CRC in response to oxaliplatin. **A** and **B** Overexpression of IMPDH2 was confirmed at the mRNA and protein level in HCT8 cells by qPCR and western blotting. **C** Comparison of apoptosis induction between vector and IMPDH2-overexpression cells in HCT8 after oxaliplatin treatment was carried out by flow cytometry analysis. Bar charts show the percentage of apoptotic and surviving cells, respectively. **D** and **E** The mRNA and protein expression levels of stable knockdown IMPDH2 in HCT8/L-OHP cells were quantified by qPCR and western blotting analysis. **F** Flow cytometry analysis was performed to analyse the apoptotic rate in shNC and shIMPDH2 HCT8/L-OHP cells after oxaliplatin treatment. **G** Western blotting analysis of apoptotic proteins expression was carried out between vector and IMPDH2-overexpression HCT8 cells after oxaliplatin treatment. **H** Western blotting analysis was performed to detect the expression of apoptotic proteins in shNC or IMPDH2-knockdown HCT8/L-OHP cells after oxaliplatin treatment. **I** The apoptotic rate was analysed after GMP and/or oxaliplatin treatment compared with that of untreated cells for 24 h by flow cytometry analysis in HCT8 cells. **J** The apoptotic rate was analysed after MPA and/or oxaliplatin treatment compared with that of untreated cells for 24 h by flow cytosmetry analysis in HCT8/L-OHP cells. Data are presented as the mean ± SD of three independent experiments. **p < 0.01, and ***p < 0.001

To further determine whether IMPDH2 regulates oxaliplatin resistance in CRC cells, we first transfected a full-length human IMPDH2 cDNA expression plasmid into CRC cells to gain of function. QPCR and western blotting analysis confirmed that IMPDH2 expression was significantly greater in HCT8 (Fig. 5A, B) and SW620 (Additional file 1: Figure S2A, B) cells than in vector-transfected cells. Flow cytometry analysis indicated that IMPDH2 overexpression decreased the proapoptotic response of CRC cells to oxaliplatin treatment. After 24 h of oxaliplatin treatment, the number of apoptotic cells was approximately twofold lower in the IMPDH2-overexpressing cells than in the vector-treated cells in the HCT8. Conversely, the percentage of surviving IMPDH2-overexpressing cells was significantly greater than that of vector-transfected HCT8 cells (Fig. 5C). Similarly, compared with IMPDH2-overexpressing SW620 cells, vector-transfected SW620 cells exhibited greater induction of cell apoptosis and a lower percentage of surviving cells (Additional file 1: Figure S2C). Next, we investigated whether IMPDH2 knockdown could promote oxaliplatin-induced apoptosis in HCT8/L-OHP cells. Reduced expression of IMPDH2 was observed at both the mRNA and protein levels in stable IMPDH2-knockdown cells (Fig. 5D, E). We found that oxaliplatin-induced apoptosis significantly increased in cells after IMPDH2 knockdown. There was also a lower number of surviving IMPDH2-knockdown cells than shNC cells after 24 h of oxaliplatin treatment (Fig. 5F). Correspondingly, we also performed a series of western blotting analysis for several apoptotic markers, including Caspase 3, Caspase 7, Caspase 8, Caspase 9, PARP1, Bax, and the corresponding active forms. As shown in Fig. 5G and Additional file 1: Figure S2E, oxaliplatin treatment led to increased expression of cleaved caspase-3, cleaved caspase-7, cleaved caspase-8, cleaved caspase-9, cleaved PARP1, and Bax in both HCT8 and SW620 cells. Overexpression of IMPDH2 resulted in reduced protein expression of cleaved caspase-7, cleaved caspase-8, cleaved caspase-9 and cleaved PARP1 in both HCT8 and SW620 cells at 24 h after oxaliplatin treatment compared to that in vector-transfected cells, but not cleaved caspase 3 or Bax. Similarly, increased expression of cleaved caspase-7, cleaved caspase-8, cleaved caspase-9 and cleaved PARP1 was detected in HCT8/L-OHP cells after IMPDH2 was knocked down (Fig. 5H). Overall, these results suggested that IMPDH2 promoted oxaliplatin resistance in CRC cells through the prevention of Caspase 7, Caspase 8, Caspase 9, and PARP1 accumulation, thereby suppressing cell apoptosis.

Considering that GMP is the metabolite most strongly associated with oxaliplatin resistance in the purine metabolic pathway (Fig. 2), we selected to exogenously add GMP substrate to HCT8 and SW620 cells to further explore the effect of oxaliplatin-induced apoptosis. Flow cytometry analysis of both HCT8 and SW620 cells revealed lower induction of cell apoptosis and a greater percentage of surviving cells in the GMP-treated groups than in the control groups (Figure 5I and Additional file 1: Figure S2D). Moreover, mycophenolic acid (MPA) inhibits GMP synthesis by blocking IMPDH; thus, we selected to inhibit GMP synthesis by MPA in HCT8/L-OHP cells. As expected, when we detected the apoptotic rate after treatment with MPA and/or oxaliplatin, MPA dramatically enhanced the proapoptotic effect of oxaliplatin in HCT8/L-OHP cells. However, the induction of cell apoptosis by MPA alone was not significantly different from that in the control group (Fig. 5J). Collectively, these results demonstrated that IMPDH2-mediated GMP synthesis regulated oxaliplatin resistance in CRC cells.

### A Wnt/β‑catenin/IMPDH2 positive feedback circuit imparts oxaliplatin resistance via GMP synthesis

**Fig. 6:**
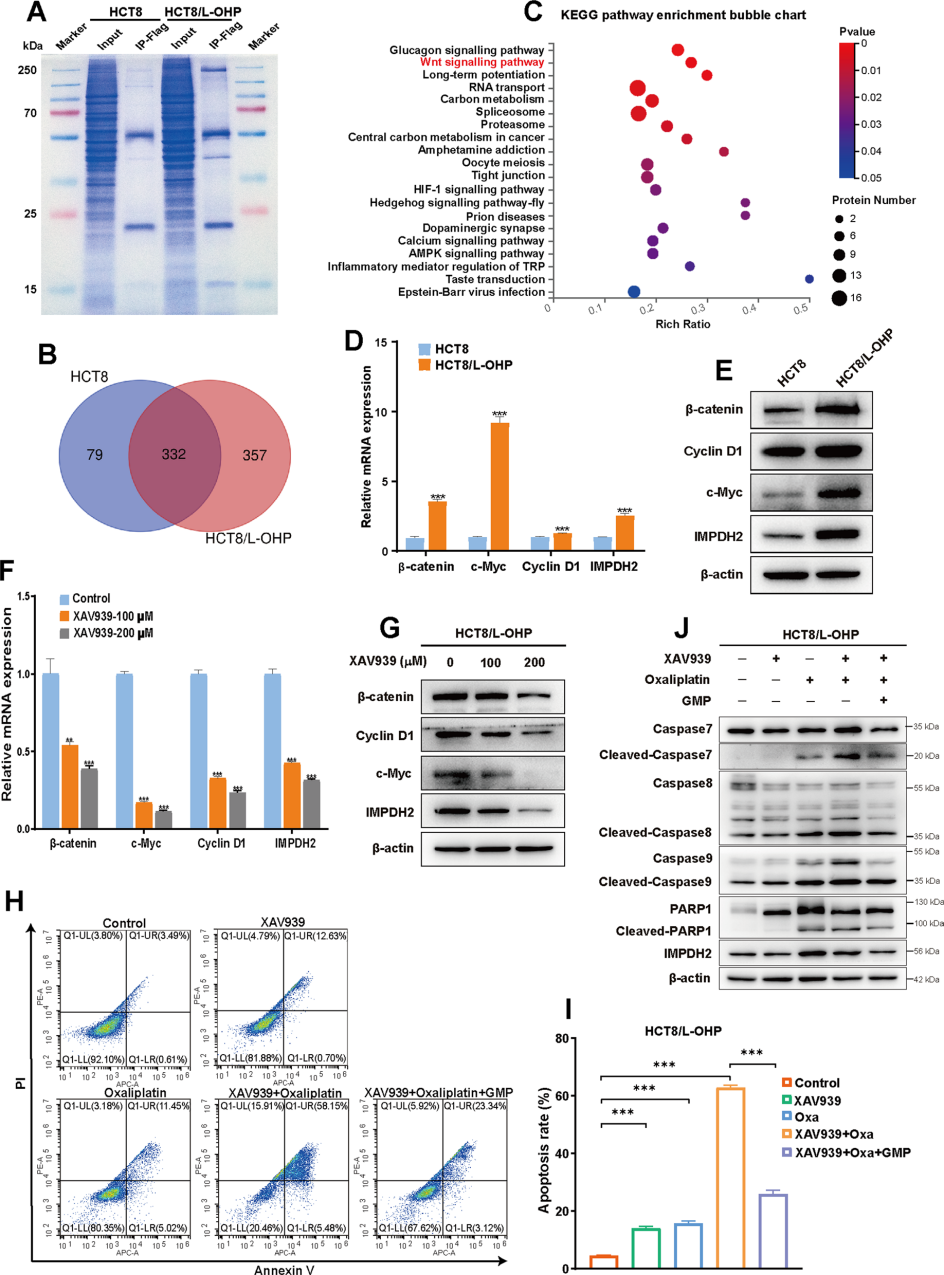
Wnt/β-catenin pathway is hyperactivated in oxaliplatin-resistant cells. **A** Coomassie staining of total proteins in HCT8 and HCT8/L-OHP cells transfecting with Flag-IMPDH2 plasmids separated by SDS–PAGE. **B** Venn diagrams displaying the number of proteins identified in HCT8 and HCT8/L-OHP cells. **C** KEGG pathway enrichment analysis of proteins identified in HCT8/L-OHP cells. **D** and **E** QPCR and western blotting analysis of the activation of the Wnt/β-catenin pathways in HCT8/L-OHP cells compared to that in HCT8 cells. **F** and **G** QPCR and western blotting analysis were conducted to evaluate the suppressive effect of XAV939 on Wnt/β-catenin pathways and IMPDH2 in HCT8/L-OHP cells. **H** and **I** The cell apoptosis was analysed after XAV939 and/or oxaliplatin treatment with or without GMP compared with that of untreated cells for 24 h by flow cytometry analysis in HCT8/L-OHP cells. **J** Western blotting analysis of apoptotic proteins expression was performed in HCT8/L-OHP cells after XAV939 and/or oxaliplatin treatment with or without GMP. Data are presented as the mean ± SD of three independent experiments. **p < 0.01, and ***p < 0.001

To elucidate the molecular signalling pathway involved in the relationship between IMPDH2-mediated GMP synthesis and oxaliplatin resistance, we performed IP-MS analysis of HCT8 and HCT8/L-OHP cells via transfection with Flag-tagged IMPDH2 plasmids (Fig. 6A). According to the LC–MS/MS results, approximately 79 specific proteins were identified in HCT8 cells, and 357 specific proteins were identified in HCT8/L-OHP cells (Fig. 6B). Furthermore, KEGG pathway enrichment analysis revealed that the specific proteins in HCT8/L-OHP cells were significantly enriched in the Wnt signalling pathway (Fig. 6C and Additional file 1: Figure S3). Given that the canonical Wnt/β-catenin pathway is the most common Wnt signalling pathway [25], we selected β-catenin, the critical molecule of the canonical Wnt/β-catenin pathway, and its well-recognized downstream target genes Cyclin D1 and c-Myc for further verification. Here, we explored the regulatory relationship between IMPDH2 and β-catenin. QPCR and western blotting analysis confirmed that the mRNA and protein expression levels of β-catenin, Cyclin D1 and c-Myc were significantly greater in HCT8/L-OHP cells than in HCT8 cells (Fig. 6D, E), indicating that the Wnt/β-catenin signalling pathway was activated in oxaliplatin-resistant cells. Next, we further explored the impact of pharmacological inhibition of β-catenin by XAV939, a well-known small molecule inhibitor that targets the poly-ADP-ribosyltransferase tankyrase 1 and 2 to destabilize β-catenin, on the expression of β-catenin, Cyclin D1, c-Myc and IMPDH2 in HCT8/L-OHP cells. We found that XAV939 substantially reduced the mRNA and protein levels of β-catenin, Cyclin D1 and c-Myc, as did IMPDH2 (Fig. 6F, G). Interestingly, overexpression or knockdown of IMPDH2 affected the Wnt/β-catenin signalling pathway. First, western blotting analysis indicated that overexpression of IMPDH2 significantly upregulated the expression levels of β-catenin, Cyclin D1 and c-Myc in both HCT8 and SW620 cells (Additional file 1: Figure S4A, B). Moreover, IMPDH2 knockdown reduced the expression levels of β-catenin, Cyclin D1 and c-Myc in HCT8/L-OHP cells (Additional file 1: Figure S4C). Collectively, these results suggested that the canonical Wnt/β-catenin pathway is hyperactivated in oxaliplatin-resistant CRC cells, and a reciprocal positive regulatory mechanism exists between Wnt/β-catenin and IMPDH2.

Since the activated Wnt/β-catenin pathway is key for oxaliplatin resistance, we further tested whether XAV939 could resensitize resistant cells to oxaliplatin. To test this hypothesis, we treated HCT8/L-OHP cells with the control, XAV939 or oxaliplatin monotherapy, or the combination of XAV939 and oxaliplatin. Flow cytometry analysis revealed that the induction of cell apoptosis by XAV939 or oxaliplatin alone was mild, while combination treatment with XAV939 and oxaliplatin significantly increased cell apoptosis. However, the increase in apoptosis was almost completely abolished when GMP was added (Fig. 6H, I). Consistently, western blotting analysis indicated that cleaved caspase 7, cleaved caspase-8, cleaved caspase-9 and cleaved PARP1 protein expression was significantly upregulated when oxaliplatin was combined with XAV939, while the expression of all of the abovementioned proteins, including IMPDH2, decreased to the level of single oxaliplatin or XAV939 treatment when GMP was rescued (Fig. 6J). Collectively, these results suggested that blocking the Wnt/β-catenin pathway could resensitize resistant cells to oxaliplatin, which could be restored by the addition of GMP.

### Inhibition of IMPDH2 reversed oxaliplatin resistance in vivo

**Fig. 7:**
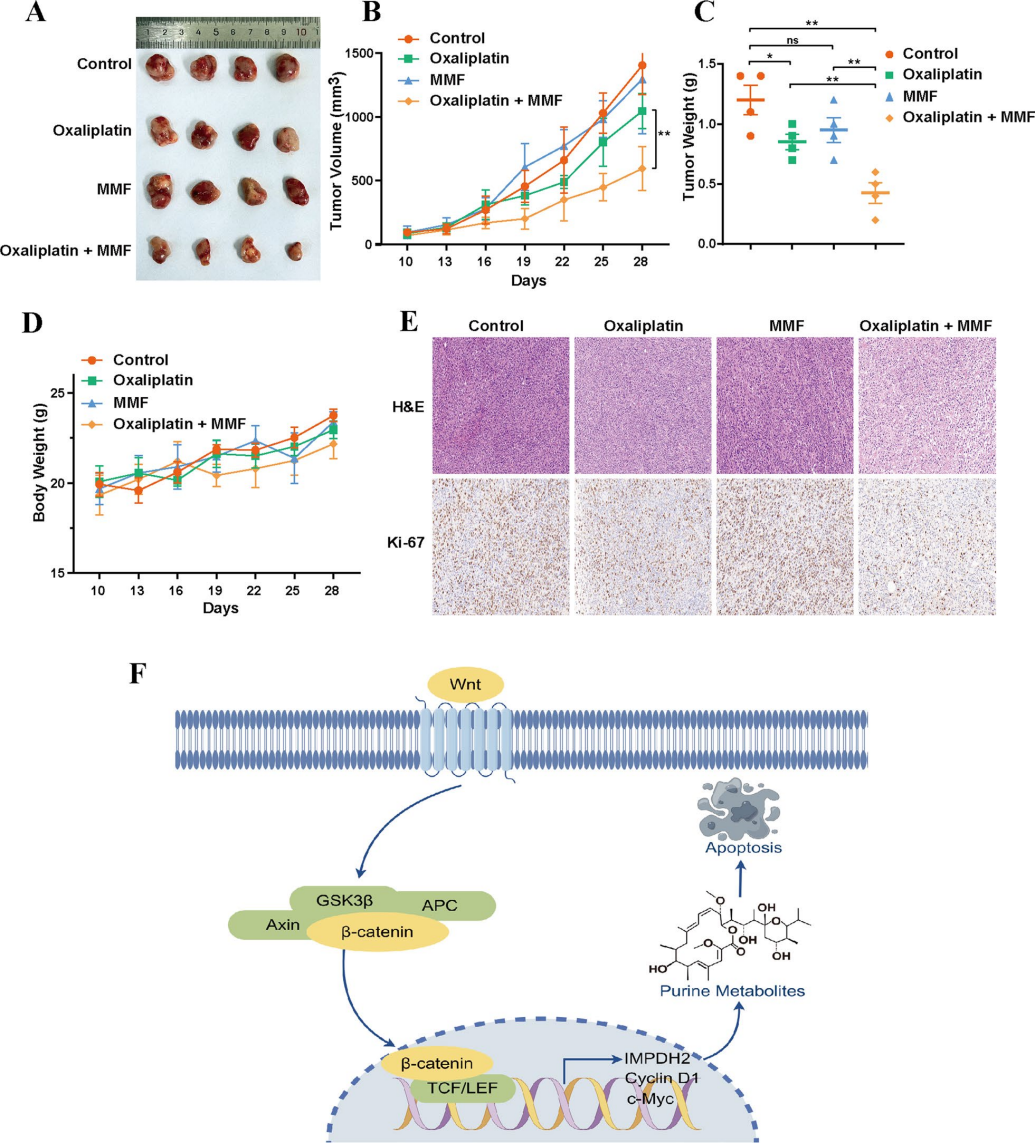
Inhibition of IMPDH2 reversed oxaliplatin resistance in vivo. **A** Tumour images. **B** Tumour growth curve. **C** Tumour weight. **D** Body weight. **E** The H&E and IHC of Ki-67 of tumour tissue with indicated treatment. **F** A schematic diagram of the Wnt/β‑catenin/IMPDH2 mediated purine metabolism regulating oxaliplatin-induced apoptosis in CRC. ns, not significant, *p < 0.05, **p < 0.01, and ***p < 0.001

Our findings suggest the potential clinical value of IMPDH2 as an intervention for oxaliplatin resistance. To further investigate whether pharmacological inhibition of IMPDH2 could reverse oxaliplatin resistance in vivo, we established subcutaneous xenograft tumour models by inoculating HCT8/L-OHP cells into the right flank of nude mice. Once the tumours were palpable, the mice were treated with control, oxaliplatin monotherapy, the IMPDH2 inhibitor MMF monotherapy, or a combination of oxaliplatin (10 mg/kg every 3 days) and MMF (120 mg/kg twice a day) for 3 weeks. At the end of treatment, tumours were harvested from the mice and examined (Fig. 7A). We observed that the monotherapy of oxaliplatin only had marginal inhibition on the tumour growth in the CRC oxaliplatin resistance model, and MMF alone did not have a sufficient therapeutic effect, while the combination treatment of oxaliplatin and MMF substantially suppressed tumour growth (Fig. 7B, C). The body weights of the experimental mice did not differ markedly (Fig. 7D). In addition, the tumours were dissected and stained with IHC to analyse the expression of the proliferation marker Ki-67. The combination treatment markedly reduced the Ki-67 expression, while monotherapy had little effect (Fig. 7E). These findings indicated that the IMPDH2 inhibitor restored the sensitivity of HCT8/L-OHP tumours to oxaliplatin in vivo.

## Discussion

Chemoresistance remains a major challenge in the management of patients with CRC. Several mechanisms of oxaliplatin resistance have been reported, including reduced cellular drug uptake and accumulation [26], increased repair of DNA damage [27], induction of epithelial-mesenchymal transition (EMT) [28], and desensitization to cell apoptosis [29]; however, effective strategies for reversing resistance have not been achieved. Emerging evidence suggests that aberrant cancer metabolism could impart drug resistance [30]. In the present study, we characterized the role of metabolic rewiring in oxaliplatin resistance and identified that purine metabolism strongly correlates with oxaliplatin resistance in CRC. The Wnt/β-catenin pathway activates IMPDH2-mediated purine metabolism to reduce oxaliplatin-induced apoptosis in CRC. We also demonstrated that knockdown and pharmacological inhibition of IMPDH2 could reverse oxaliplatin resistance in vivo and in vitro.

Aberrant cancer metabolism is considered one of the important hallmarks of cancer cells [31]. Generally, rapidly proliferating cancer cells require both elevated energy consumption and increased nucleotide biosynthetic pathways to sustain high levels of DNA replication and RNA transcription [32]. Purine metabolism is the most important source of nucleotide biosynthesis, and increased purine metabolites can accelerate cancer cell proliferation and tumour progression [33]. Targeting IMPDH, a key regulator of purine metabolism, effectively suppressed hepatocellular carcinoma progression [34]. Another study showed that purine metabolites, especially guanylates, strongly correlate with radiation resistance in glioblastoma and that inhibiting GTP synthesis radiosensitizes glioblastoma cells [15]. These studies imply that inhibiting key rate-limiting enzymes or reducing intermediate metabolites of purine metabolism may be attractive strategies for cancer therapy. Consistently, using an untargeted metabolomics approach, we identified that the metabolic levels of purine metabolism products, especially GMP, a vital intermediate metabolite, were significantly elevated in oxaliplatin-resistant cells. We further confirmed that the sensitivity of cells to oxaliplatin decreased when GMP was exogenously supplemented in oxaliplatin-sensitive CRC cells. However, the inhibition of GMP synthesis by MPA or MMF, which are currently available clinical therapeutic drugs, impaired oxaliplatin resistance, led to significant cell death in CRC cells in vitro, and even suppressed xenograft tumour growth in nude mice.

Interestingly, oxaliplatin cytotoxicity has been primarily ascribed to interactions between oxaliplatin and DNA that can form DNA adducts that lead to cell cycle arrest and apoptosis [35]. Mechanistically, purine metabolites provide an important substrate pool for DNA replication and RNA transcription to fuel the biosynthetic demands of cell growth and division. Activated purine metabolism, especially GMP, can reverse DNA damage caused by oxaliplatin, thus contributing to resistance in CRC. IMPDH is one of the major checkpoints for the activity of purine metabolism; this enzyme catalyses the transformation of inosine monophosphate (IMP) to XMP, which is further converted into GTP for DNA and RNA synthesis [14]. Inhibition of IMPDH results in decreased cellular guanine nucleotide pools and subsequently suppresses the synthesis of DNA and RNA, thus inhibiting cancer cell proliferation by blocking cell cycle progression and inducing cell apoptosis [36]. Accumulating evidence suggests that IMPDH2 is the predominant isoform of IMPDH, and increased IMPDH2 expression is associated with tumorigenesis, metastasis and recurrence in most cancers, including leukaemia [37], prostate cancer [38], ovarian cancer [39], non-small cell lung cancer [40], and triple-negative breast cancer [41]. In particular, Duan et al. reported that IMPDH2 is highly expressed in CRC and is correlated with poor survival in CRC patients, and the overexpression of IMPDH2 dramatically promoted CRC progression [13]. Herein, we found that both IMPDH1 and IMPDH2 were significantly upregulated in oxaliplatin-resistant CRC cells, whereas the increase in IMPDH2 was twofold greater than that in IMPDH1. Further studies revealed that CRC cells with higher IMPDH2 expression were more resistant to oxaliplatin-induced apoptosis. Overexpression of IMPDH2 in CRC cells resulted in reduced cell death upon treatment with oxaliplatin, whereas knockdown of IMPDH2 led to increased sensitivity to oxaliplatin. Western blotting analysis demonstrated that IMPDH2 inhibited cell apoptosis through the prevention of the accumulation of cleaved caspase 7, caspase 8, caspase 9, and PARP1, thereby promoting resistance to oxaliplatin in CRC cells. Moreover, the combination of the IMPDH2 inhibitor MPA or the addition of the GMP substrate with oxaliplatin produced similar results. Thus, we hypothesize that purine reprogramming is a novel mechanism of oxaliplatin resistance and that inhibiting purine metabolism by targeting the key enzyme IMPDH2 may be a potential therapeutic approach for reversing oxaliplatin resistance in CRC.

The Wnt/β-catenin signalling pathway not only plays an essential role in tumorigenesis and cancer progression [17], but is also associated with chemotherapeutic resistance in multiple cancers [21, 42]. Additionally, aberrant activation of Wnt/β-catenin signalling could not only directly induce glucose metabolic reprogramming and promote colon cancer cell proliferation [23] but also indirectly activate downstream transcription factors such as HIF-1ɑ to induce metabolic reprogramming and impart 5-fluorouracil resistance in CRC [42]. In the present study, we discovered that the Wnt/β‑catenin signalling pathway is hyperactivated in oxaliplatin-resistant CRC cells. The activated Wnt/β‑catenin signalling pathway promote IMPDH2 expression, whose increase, in turn, induced the expression of β‑catenin, forming a Wnt/β-catenin/IMPDH2 positive feedback circuit that confers resistance to oxaliplatin in CRC. We further performed flow cytometry analysis, and the results showed that the combination of XAV939, a tankyrase inhibitor that targets Wnt/β-catenin, with oxaliplatin markedly increased cell apoptosis when compared with XAV939 or oxaliplatin alone; however, these effects were nearly reversed by the addition of GMP. Taken together, our study revealed that the Wnt/β‑catenin signalling pathway may be a master regulator of purine metabolic reprogramming in oxaliplatin resistance. However, c-Myc also serves as an oncogene that directly induces enhanced expression of target enzymes involved in the metabolism of a variety of enzymes, including IMPDH2 [43]. Thus, the mechanism underlying the interaction between c-Myc and the Wnt/β‑catenin pathway and IMPDH2 in CRC oxaliplatin resistance remains to be assessed in our future research. Additionally, tumour-infiltrating immune cells (TIICs) play significant roles in chemotherapeutic resistance in rectal cancer [44]. MMF has been widely approved as an immunosuppressant in organ transplant recipients and can also mediate the tumour microenvironment [45]. Therefore, it is reasonable to assume that MMF, or targeting IMPDH2, may exert anticancer activity via its immunosuppressive effects on TIICs, but further studies are needed.

## Conclusions

In conclusion, our study suggested that purine metabolism strongly correlates with oxaliplatin resistance in CRC and that a Wnt/β‑catenin/IMPDH2 positive feedback circuit confers oxaliplatin resistance (Fig. 7F). Targeting IMPDH2 by gene knockdown and pharmacological inhibition could reverse oxaliplatin resistance in vivo and in vitro. Our findings support the concept that IMPDH2 is a potential biomarker for oxaliplatin resistance and a therapeutic target for overcoming oxaliplatin resistance in CRC.

### Supplementary Information

**Additional file 1: Figure S1.** Network diagram of purine metabolic pathway. Small circle: metabolite (the red indicates that the metabolite is up-regulated, while the blue indicates that the metabolite is down-regulated in the comparison group); arrow: direction of response; small box: enzyme; large box: other metabolic pathways. **Figure S2. **IMPDH2 regulates CRC cell apoptosis in response to oxaliplatin. **A** and **B** Overexpression of IMPDH2 was confirmed at the mRNA and protein level in SW620 cells by qPCR and western blotting analysis. **C** Comparison of apoptosis induction between vector and IMPDH2-overexpression cells in SW620 after oxaliplatin treatment was carried out by flow cytometry analysis. Bar charts show the percentage of apoptotic and surviving cells, respectively. **D** The apoptotic rate was analysed after GMP and/or oxaliplatin treatment compared with that of untreated cells for 24 h by flow cytometry analysis in SW620 cells. **E** Western blotting analysis of apoptotic proteins expression was carried out between vector and IMPDH2-overexpression SW620 cells after oxaliplatin treatment. **p < 0.01, and ***p < 0.001. **Figure S3**. Network diagram of the Wnt signalling pathway. The small purple rectangle represents the uniquely proteins identified in HCT8/L-OHP cells.

## Data Availability

The datasets used during the current study are available from the corresponding author on reasonable request.
