# Supplementary material for: Wnt/β-catenin signalling activates IMPDH2-mediated purine metabolism to facilitate oxaliplatin resistance by inhibiting caspase-dependent apoptosis in colorectal cancer
Source: J Transl Med. 2024 Feb 3;22:133. doi: 10.1186/s12967-024-04934-0 (PMC10838440; doi:10.1186/s12967-024-04934-0)
Supplement: Supplementary file 1 — Additional file 1: Figure S1. Network diagram of purine metabolic pathway. Small circle: metabolite (the red indicates that the metabolite is up-regulated, while the blue indicates that the metabolite is down-regulated in the comparison group); arrow: direction of response; small box: enzyme; large box: other metabolic pathways. Figure S2. IMPDH2 regulates CRC cell apoptosis in response to oxaliplatin. A and B Overexpression of IMPDH2 was confirmed at the mRNA and protein level in SW620 cells by qPCR and western blotting analysis. C Comparison of apoptosis induction between vector and IMPDH2-overexpression cells in SW620 after oxaliplatin treatment was carried out by flow cytometry analysis. Bar charts show the percentage of apoptotic and surviving cells, respectively. D The apoptotic rate was analysed after GMP and/or oxaliplatin treatment compared with that of untreated cells for 24 h by flow cytometry analysis in SW620 cells. E Western blotting analysis of apoptotic proteins expression was carried out between vector and IMPDH2-overexpression SW620 cells after oxaliplatin treatment. **p < 0.01, and ***p < 0.001. Figure S3. Network diagram of the Wnt signalling pathway. The small purple rectangle represents the uniquely proteins identified in HCT8/L-OHP cells. [file 12967_2024_4934_MOESM1_ESM.docx]

**
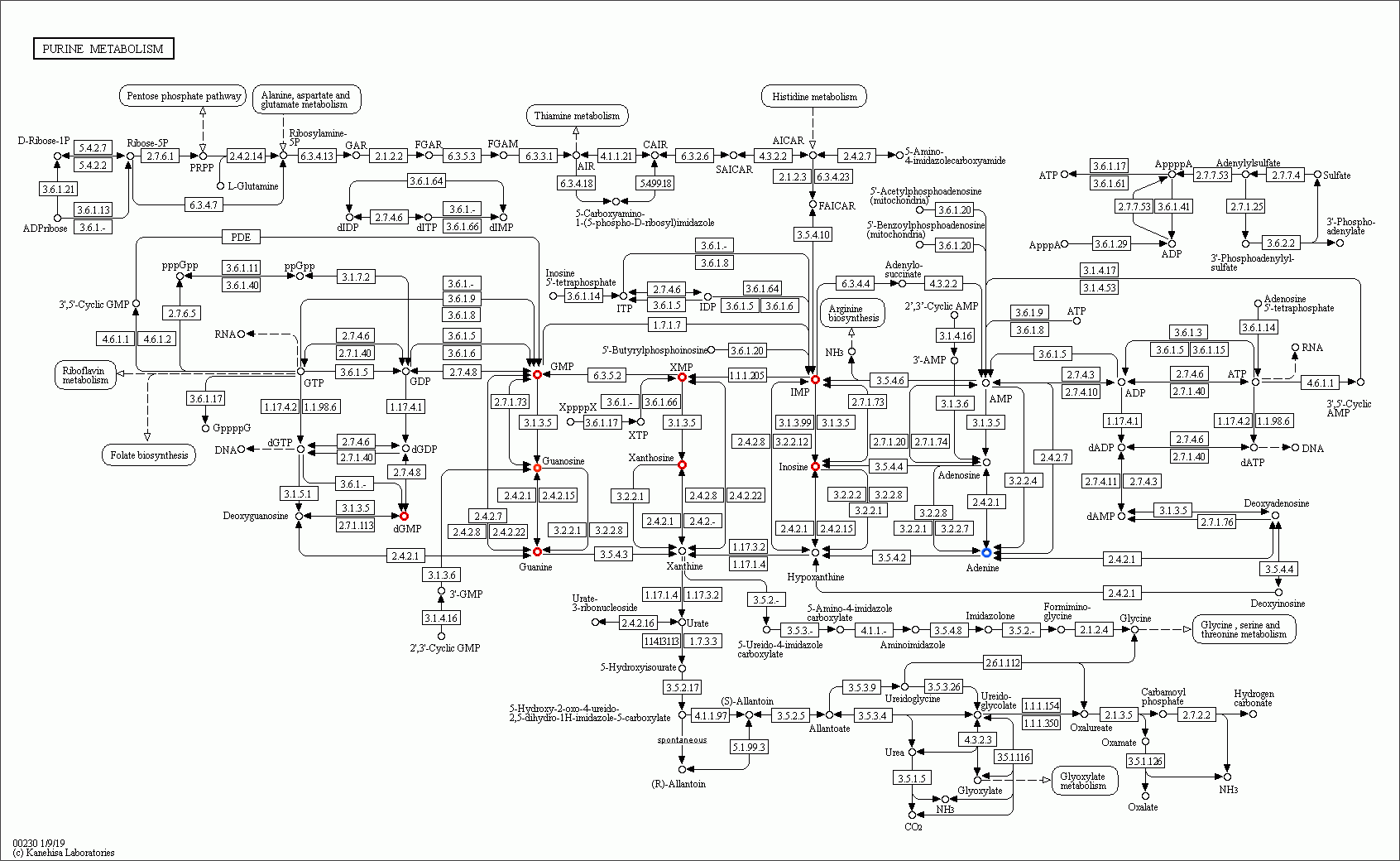
**

**Supplemental Figure S1. Network diagram of purine metabolic pathway.** Small circle: metabolite (the red indicates that the metabolite is up-regulated, while the blue indicates that the metabolite is down-regulated in the comparison group); arrow: direction of response; small box: enzyme; large box: other metabolic pathways.


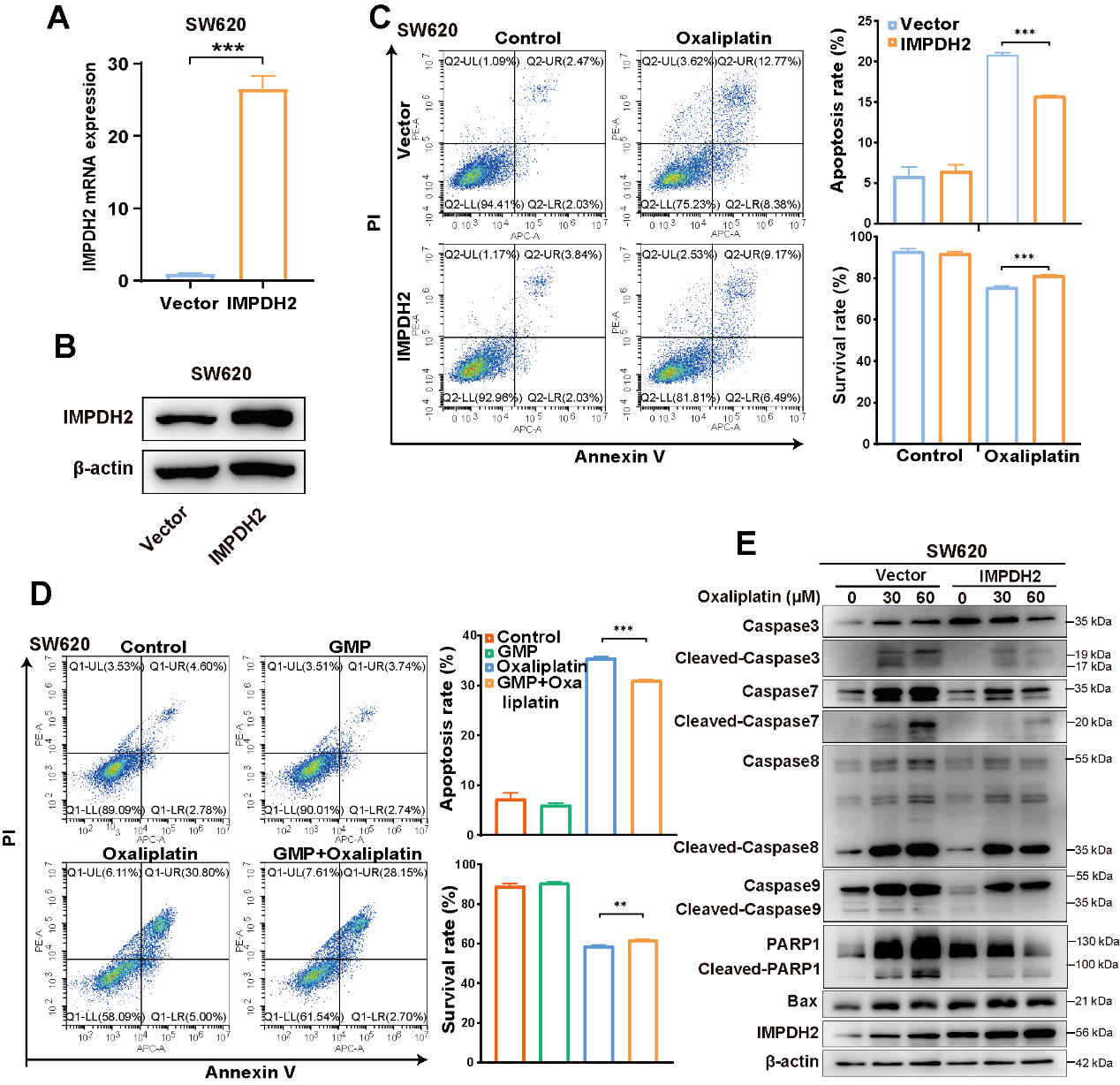


**Supplemental Figure S2. IMPDH2 regulates CRC cell apoptosis in response to oxaliplatin. (A and B)** Overexpression of IMPDH2 was confirmed at the mRNA and protein level in SW620 cells by qPCR and western blotting analysis. **(C)** Comparison of apoptosis induction between vector and IMPDH2-overexpression cells in SW620 after oxaliplatin treatment was carried out by flow cytometry analysis. Bar charts show the percentage of apoptotic and surviving cells, respectively. **(D)** The apoptotic rate was analysed after GMP and/or oxaliplatin treatment compared with that of untreated cells for 24 h by flow cytometry analysis in SW620 cells. **(E)** Western blotting analysis of apoptotic proteins expression was carried out between vector and IMPDH2-overexpression SW620 cells after oxaliplatin treatment. ***p* < 0.01, and ****p* < 0.001.


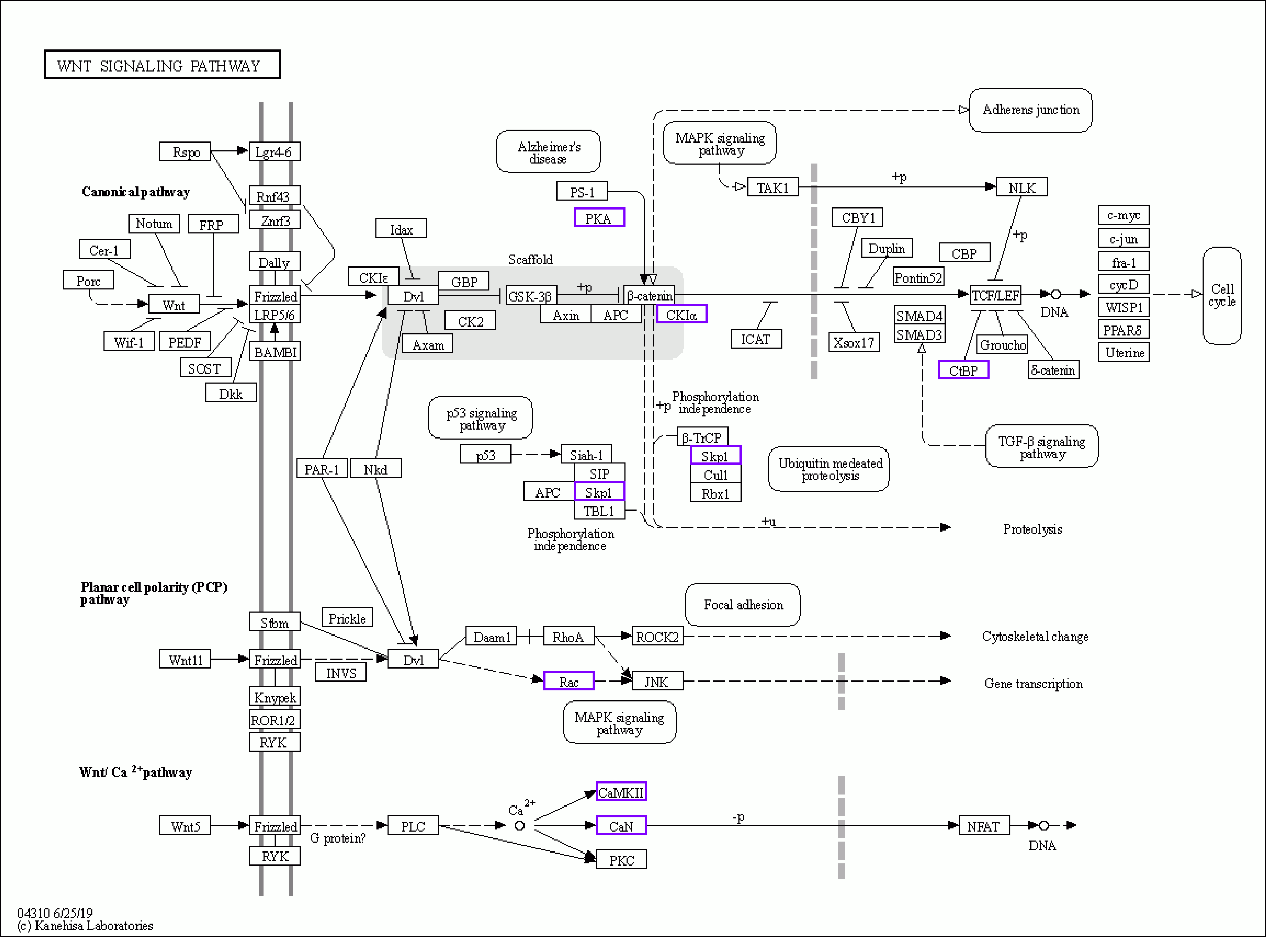


**Supplementary Figure S3**. **Network diagram of the Wnt signalling pathway.** The small purple rectangle represents the uniquely proteins identified in HCT8/L-OHP cells.
